# Supplementary material for: Mostly natural sequencing-by-synthesis for scRNA-seq using Ultima sequencing
Source: Nat Biotechnol. 2022 Sep 15;41(2):204–11. doi: 10.1038/s41587-022-01452-6 (PMC9931582; doi:10.1038/s41587-022-01452-6)
Supplement: Supplementary file 1 — Supplementary Figs. 1–3 and Supplementary Tables 1, 3, 4 and 6. [file 41587_2022_1452_MOESM1_ESM.pdf]

nature portfolio

https://doi.org/10.1038/s41587-022-01452-6

Supplementary information

Mostly natural sequencing-by-synthesis for scRNA-seq using Ultima sequencing

In the format provided by the authors and unedited

Supp. Fig. 1

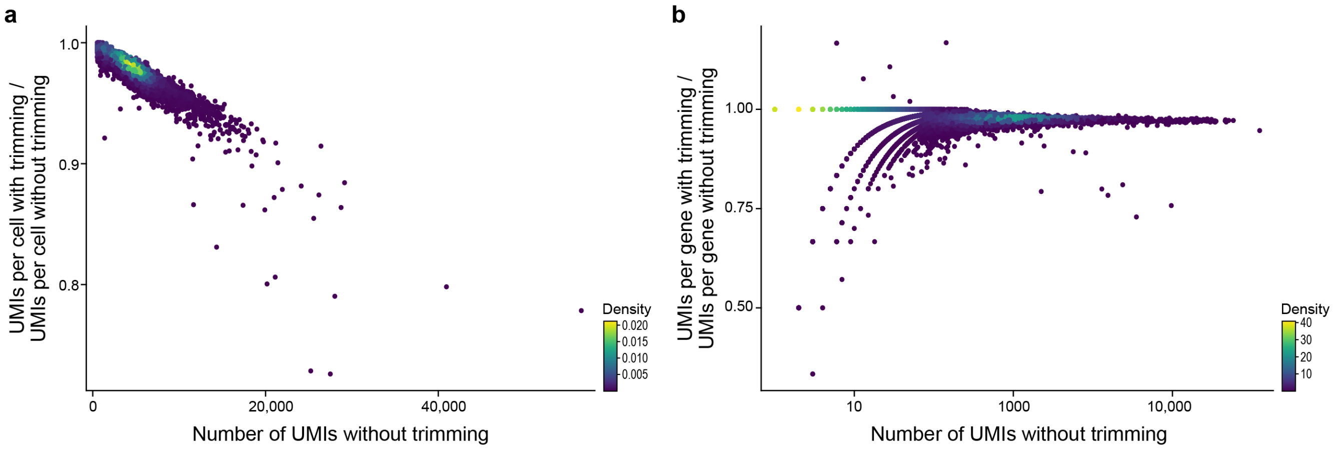

Supplementary Figure 1. Relationship between UMI coverage and UMI trimming.

The number of UMIs per (**a**) cell and (**b**) gene for 3' PBMC Illumina data with both full-length UMIs and UMIs trimmed to 9 bases. Ratio between the two (the number of UMIs per cell or gene with trimmed UMIs divided by the number of UMIs per cell or gene with untrimmed UMIs) is plotted versus the number of UMIs without trimming. Each point is colored by its local density in the graph.

Supp. Fig. 2

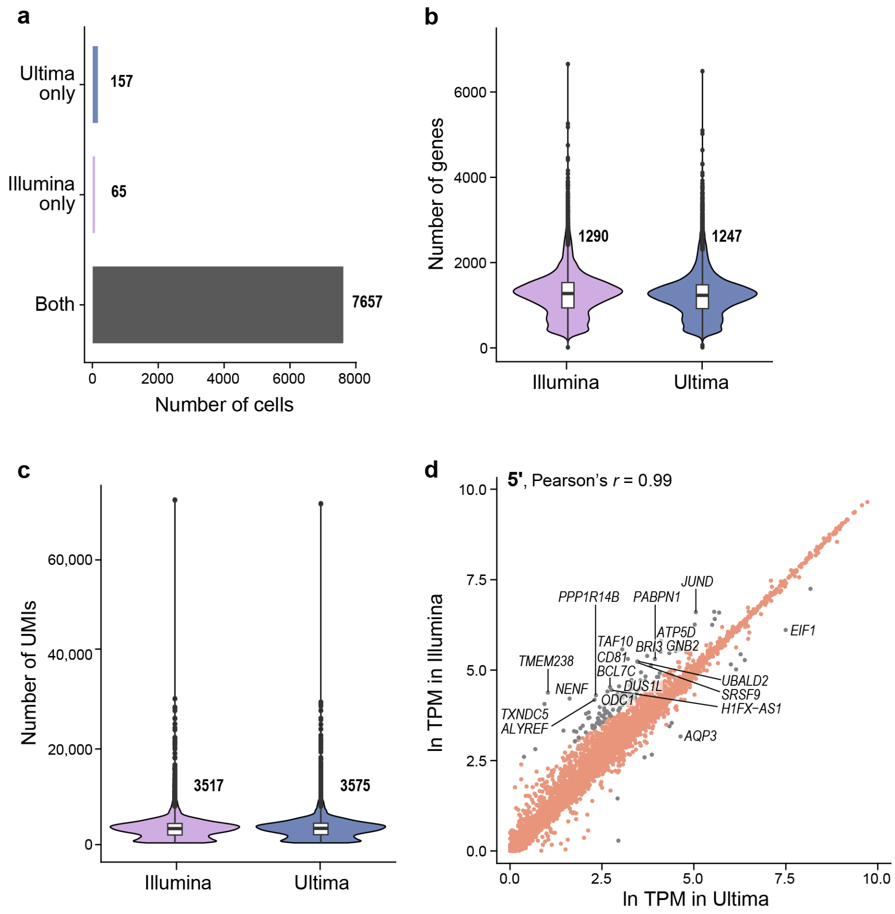

Supplementary Figure 2. Quality metrics for 5' Ultima vs single-end Illumina data. (**a**) Number of cells identified by Cell Ranger only in Ultima, only in Illumina, or both. Distribution of the number of genes (**b**) or UMIs (**c**) per cell. (**d**) Scatter plots with one point for each gene as in **Fig. 2e**. For all these analyses, reads were sampled so that Illumina and Ultima have the same number of reads.

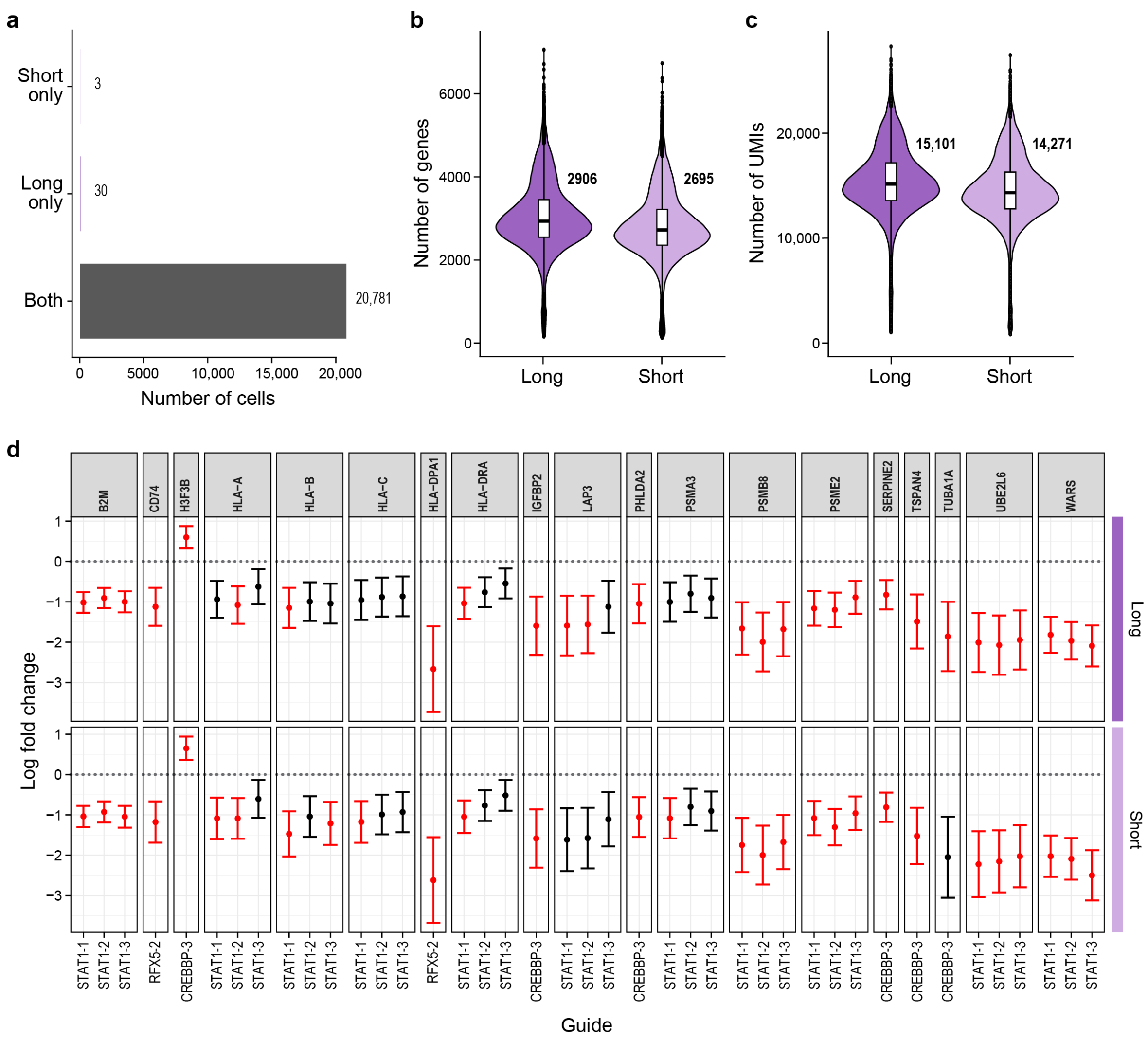

Supplementary Figure 3. Perturb-seq data with shortened Read 2 length.

(**a**) Number of cells identified only in the shorter Illumina data, only in longer Illumina data, or in both. Distribution of the number of genes (**b**) or UMIs (**c**) per cell in short and long Illumina data. (**d**) We extracted all gene/guide pairs from our DE analysis with FDR < 0.05 in either shorter or longer Illumina data. Data processed and shown here as in **Fig. 4f**.

Supplementary Tables

Supplementary Table 1. Sequencing metrics.

Supplementary Table 2. Comparison of read mapping between Ultima and Illumina.

Supplementary Table 3. Differentially expressed genes between Ultima and Illumina with standard or extended references.

Supplementary Table 4. Antibody, Perturb-seq, and hashing DNA barcodes.

Supplementary Table 5. PCR primers to convert libraries for Ultima sequencing.

Supplementary Table 6. Commands for initial sequence processing.

Name	Sequencer	UMI Length	Library	Removed UMIs with base quality < 10	Sampling (equal # of)	Read2 Length	Reference	Estimated Number of Cells	Mean Reads per Cell	Median Genes per Cell	Number of Reads	Valid Barcodes	Sequencing Saturation	Q30 Bases in Barcode	Q30 Bases in RNA Read	Q30 Bases in UMI	Reads Mapped to Genome	Reads Mapped Confidently to Genome	Reads Mapped Confidently to Intergenic Regions	Reads Mapped Confidently to Intronic Regions	Reads Mapped Confidently to Exonic Regions	Reads Mapped Confidently to Transcriptome	Reads Mapped Antisense to Gene	Fraction Reads in Cells	Total Genes Detected	Median UMI Counts per Cell
threeprime_umi_ill	Illumina	9	3' PBMC	No	none	55	Standard	7,923	19,702	1,348	156,100,101	98%	51%	95%	92%	96%	86%	82%	4%	24%	54%	51%	1%	93%	21,810	4,165
threeprime_noumi_ill	Illumina	12	3' PBMC	No	none	55	Standard	7,926	19,694	1,366	156,100,101	98%	51%	95%	92%	95%	86%	82%	4%	24%	54%	51%	1%	93%	21,892	4,259
threeprime_umi_ult	Ultima	9	3' PBMC	No	none	90	Standard	8,100	49,327	1,415	399,549,702	97%	72%	94%	83%	85%	97%	93%	6%	33%	55%	52%	1%	93%	20,586	5,068
threeprime_umi_UMIDS_ult	Ultima	9	3' PBMC	No	UMIs	90	Standard	8,015	29,411	1,268	235,737,147	97%	61%	94%	83%	85%	97%	93%	6%	33%	55%	52%	1%	93%	20,173	4,193
threeprime_umi_ReadDS_ult	Ultima	9	3' PBMC	No	reads	90	Standard	7,916	19,683	1,128	155,813,953	97%	51%	94%	83%	85%	97%	93%	6%	33%	55%	52%	1%	93%	19,786	3,507
threeprime_umi_short_ult	Ultima	9	3' PBMC	No	none	55	Standard	8,094	49,363	1,421	399,549,702	97%	72%	94%	84%	85%	96%	89%	5%	30%	54%	52%	1%	93%	20,894	5,052
threeprime_noumi_filt_ult	Ultima	12	3' PBMC	Yes	none	90	Standard	8,094	34,081	1,364	275,851,725	97%	64%	95%	84%	77%	97%	93%	6%	32%	55%	52%	1%	93%	20,454	5,418
threeprime_noumi_ult	Ultima	12	3' PBMC	No	none	90	Standard	8,119	49,211	1,379	399,549,702	97%	64%	94%	83%	74%	97%	93%	6%	33%	55%	52%	1%	93%	20,505	5,550
threeprime_umi_bulkext_ult	Ultima	9	3' PBMC	No	reads	90	Bulk	7,985	19,513	1,166	155,813,953	97%	51%	94%	83%	85%	97%	93%	5%	31%	57%	54%	1%	93%	19,907	3,648
threeprime_umi_bulkext_ill	Illumina	9	3' PBMC	No	none	55	Bulk	7,936	19,669	1,426	156,100,101	98%	50%	95%	92%	95%	86%	81%	4%	23%	55%	52%	1%	93%	21,983	4,340
threeprime_umi_scext_ult	Ultima	9	3' PBMC	No	reads	90	3' SC	7,972	19,545	1,166	155,813,953	97%	51%	94%	83%	85%	97%	93%	6%	32%	56%	54%	1%	93%	20,603	3,627
threeprime_umi_scext_ult	Illumina	9	3' PBMC	No	none	55	3' SC	7,930	19,684	1,382	156,100,101	98%	51%	95%	92%	95%	86%	82%	4%	24%	54%	52%	1%	93%	21,958	4,288
fiveprime_noumi_ill	Illumina	10	5' PBMC	No	none	45	Standard	7,854	24,008	1,374	188,562,544	88%	66%	94%	85%	91%	91%	67%	3%	8%	56%	51%	3%	93%	22,082	3,612
fiveprime_ill_SE	Illumina (SE)	10	5' PBMC	No	none	90	Standard	7,722	20,292	1,287	156,702,072	99%	70%	99%	85%	98%	99%	83%	4%	7%	72%	64%	3%	93%	19,609	3,503
fiveprime_all_ult	Ultima	10	5' PBMC	No	none	90	Standard	8,151	70,759	1,480	576,757,306	96%	88%	97%	89%	95%	99%	83%	4%	9%	70%	63%	3%	93%	20,557	4,418
fiveprime_UMIDS_ult	Ultima	10	5' PBMC	No	UMIs	90	Standard	7,829	20,627	1,249	161,493,951	96%	68%	97%	89%	95%	99%	83%	4%	9%	70%	63%	3%	93%	19,786	3,566
fiveprime_ReadDS_ult	Ultima	10	5' PBMC	No	reads	90	Standard	7,875	23,436	1,282	184,561,342	96%	71%	97%	89%	95%	99%	83%	4%	9%	70%	63%	3%	93%	19,868	3,681
fiveprime_short_ult	Ultima	10	5' PBMC	No	none	45	Standard	8,175	70,551	1,486	576,757,306	96%	88%	97%	90%	95%	98%	81%	4%	8%	69%	63%	3%	93%	21,346	4,379
fiveprime_bulkext_ult	Ultima	10	5' PBMC	No	reads	90	Bulk	7,958	23,191	1,347	184,561,342	96%	71%	97%	89%	95%	99%	83%	3%	8%	72%	66%	3%	93%	19,995	3,803
fiveprime_bulkext_ill	Illumina	10	5' PBMC	No	none	45	Bulk	7,900	23,868	1,410	188,562,544	88%	66%	94%	85%	91%	91%	66%	3%	7%	57%	52%	4%	93%	22,133	3,650
fiveprime_scext_ult	Ultima	10	5' PBMC	No	reads	90	3' SC	7,979	23,130	1,367	184,561,342	96%	71%	97%	89%	95%	99%	83%	4%	8%	71%	66%	3%	93%	20,975	3,819
fiveprime_scext_ult	Illumina	10	5' PBMC	No	none	45	3' SC	7,894	23,886	1,391	188,562,544	88%	66%	94%	85%	91%	91%	67%	3%	7%	57%	52%	3%	93%	22,147	3,637
fiveprime_mix_ill	Illumina	10	5' PBMC Mixture	No	none	45	Standard	26,923	27,431	1,578	738,535,623	88%	60%	92%	84%	89%	89%	70%	4%	12%	53%	46%	5%	88%	25,815	3,694
fiveprime_mix_ult	Ultima	10	5' PBMC Mixture	No	none	90	Standard	27,156	28,840	1,385	783,197,657	94%	66%	94%	81%	91%	96%	84%	7%	15%	62%	53%	5%	88%	23,667	3,563
perturbseq_noumi_ill	Illumina	12	3' Perturb-seq	No	none	96	Standard	20,811	18,926	2,905	393,889,367	98%	10%	97%	92%	97%	97%	94%	8%	17%	69%	65%	2%	97%	24,739	9,452
perurbseq_ill_short	Illumina	12	3' Perturb-seq	No	none	55	Standard	20,784	18,951	2,694	393,889,367	98%	10%	97%	93%	97%	82%	77%	4%	13%	60%	58%	1%	97%	25,097	8,444
perturbseq_umi_ult	Ultima	9	3' Perturb-seq	No	none	90	Standard	21,168	45,795	3,785	969,406,534	98%	24%	95%	87%	85%	98%	95%	8%	19%	68%	64%	1%	97%	24,683	15,668
perturbseq_umi_UMIDS_ult	Ultima	9	3' Perturb-seq	No	UMIs	90	Standard	20,936	25,005	2,896	523,508,222	98%	15%	95%	87%	85%	98%	95%	8%	19%	68%	64%	1%	97%	23,777	9,769
	SE=Single-end																									

Table S3

Method	Illumina read type	Illumina Reference	Ultima Reference**	Total outliers*	Outliers upregulated in Illumina	Outliers upregulated in Ultima
3'	Paired-end	Standard	Standard	182	131	51
3'	Paired-end	Standard	Extended by single cell	173	118	55
3'	Paired-end	Standard	Extended by bulk	177	117	60
3'	Paired-end	Extended by single cell	Extended by single cell	172	119	53
3'	Paired-end	Extended by bulk	Extended by bulk	183	135	48
5'	Paired-end	Standard	Standard	454	342	112
5'	Paired-end	Standard	Extended by single cell	352	233	119
5'	Paired-end	Standard	Extended by bulk	350	234	116
5'	Paired-end	Extended by single cell	Extended by single cell	346	247	109
5'	Paired-end	Extended by bulk	Extended by bulk	349	247	102
5'	Single-end	Standard	Standard	122	109	13

*Outliers are genes with absolute logFC>log(2) in Ultima relative to Illumina

**Uses Ultima data downsampled to have the same number of reads as Illumina

table S5

Name	Sequence	Notes
PS-SBC	5’-CCATCTCATCCCTGCGTGTCTCCGACTCAGCGGAAGAACCTCGATCTACACGACGCTCTTCCGATC*T-3’	IA region, Read 1 binding region
PB	5’-CCTATCCCCTGTGTGCCTTGGCAGTCTCAGCAGACGTGTGCTCTTCCGATC*T-3’	UBA region, Read 2 binding region
* = phosphorothioate bond modification protecting it from degradation		

Table S6

<table>
<tr><td>3' Ultima step 1</td><td>cutadapt -j 0 --discard-untrimmed --pair-filter any -a CTACACGACGCTCTTCCGATCT;max_error_rate=0.2;min_overlap=10;required...AGATCGGAAGAGCACACGTCTG;max_error_rate=0.2;min_overlap=6 -U 50 -q 30 -A TTTTTTTTTTTT;max_error_rate=0.2;min_overlap=8;required...AGATCGGAAGAGCACACGTCTG;max_error_rate=0.2;min_overlap=6 -o <output_read1_long> -p <output_read2> --minimum-length 28:50 <input_fastq> <input_fastq></td></tr>
<tr><td>3' Ultima step 2</td><td>cutadapt -j 0 --minimum-length 28 --maximum-length 28 --length 28 -o <output_read1> <output_read1_long></td></tr>
<tr><td>3' Ultima step 3</td><td>zcat <output_read2> | awk '{if ((NR%4 == 2) || (NR%4 == 0)) {print substr($0,1+5,90) } else {print $0 } }' | seqkit -j 8 seq -p -r -t DNA | gzip > <output_read2_revcom></td></tr>
<tr><td></td><td></td></tr>
<tr><td>5' Ultima step 1</td><td>cutadapt -j 0 --discard-untrimmed --pair-filter any -a CTACACGACGCTCTTCCGATCT;max_error_rate=0.2;min_overlap=10;required...AGATCGGAAGAGCACACGTCTG;max_error_rate=0.2;min_overlap=6 -U 48 -q 30 -A ^TTTCTTATATGGG;max_error_rate=0.5;min_overlap=8;required...AGATCGGAAGAGCACACGTCTG;max_error_rate=0.2;min_overlap=6 -o <output_read1_long> -p <output_read2> --minimum-length 26:50 --maximum-length 390:315 <input_fastq> <input_fastq></td></tr>
<tr><td>5' Ultima step 2</td><td>cutadapt -j 0 --minimum-length 26 --maximum-length 26 --length 26 -o <output_read1> <output_read1_long></td></tr>
<tr><td>5' Ultima step 3</td><td>zcat <output_read2> | awk '{if ((NR%4 == 2) || (NR%4 == 0)) {print substr($0,1+3,90) } else {print $0 } }' | seqkit -j 8 seq -p -r -t DNA | gzip > <output_read2_revcom></td></tr>
<tr><td></td><td></td></tr>
<tr><td>5' Illumina single-end step 1</td><td>cutadapt -j 0 --discard-untrimmed --pair-filter any -a CTACACGACGCTCTTCCGATCT;max_error_rate=0.2;min_overlap=10;required...AGATCGGAAGAGCACACGTCTG;max_error_rate=0.2;min_overlap=6 -U 48 -q 30 -A ^TTTCTTATATGGG;max_error_rate=0.5;min_overlap=8;required...AGATCGGAAGAGCACACGTCTG;max_error_rate=0.2;min_overlap=6 -o <output_read1_long> -p <output_read2> --minimum-length 26:50 --maximum-length 390:315 <input_fastq> <input_fastq></td></tr>
<tr><td>5' Illumina single-end step 2</td><td>cutadapt -j 0 --minimum-length 26 --maximum-length 26 --length 26 -o <output_read1> <output_read1_long></td></tr>
<tr><td>5' Illumina single-end step 3</td><td>zcat <output_read2> | awk '{if ((NR%4 == 2) || (NR%4 == 0)) {print substr($0,1+3,90) } else {print $0 } }' | seqkit -j 8 seq -p -r -t DNA | gzip > <output_read2_revcom></td></tr>
</table>
