# Supplementary material for: Mostly natural sequencing-by-synthesis for scRNA-seq using Ultima sequencing
Source: Nat Biotechnol. 2022 Sep 15;41(2):204–11. doi: 10.1038/s41587-022-01452-6 (PMC9931582; doi:10.1038/s41587-022-01452-6)
Supplement: Supplementary file 2 — Reporting Summary [file 41587_2022_1452_MOESM2_ESM.pdf]

nature portfolio

Corresponding author(s): Joshua Z. Levin

Last updated by author(s): Jul 18, 2022

Reporting Summary

Nature Portfolio wishes to improve the reproducibility of the work that we publish. This form provides structure for consistency and transparency in reporting. For further information on Nature Portfolio policies, see our Editorial Policies and the Editorial Policy Checklist.

Statistics

For all statistical analyses, confirm that the following items are present in the figure legend, table legend, main text, or Methods section.

n/a	Confirmed	
☐	☒	The exact sample size (*n*) for each experimental group/condition, given as a discrete number and unit of measurement
☐	☒	A statement on whether measurements were taken from distinct samples or whether the same sample was measured repeatedly
☐	☒	The statistical test(s) used AND whether they are one- or two-sided<br>*Only common tests should be described solely by name; describe more complex techniques in the Methods section.*
☐	☒	A description of all covariates tested
☐	☒	A description of any assumptions or corrections, such as tests of normality and adjustment for multiple comparisons
☐	☒	A full description of the statistical parameters including central tendency (e.g. means) or other basic estimates (e.g. regression coefficient) AND variation (e.g. standard deviation) or associated estimates of uncertainty (e.g. confidence intervals)
☐	☒	For null hypothesis testing, the test statistic (e.g. *F*, *t*, *r*) with confidence intervals, effect sizes, degrees of freedom and *P* value noted<br>*Give P values as exact values whenever suitable.*
☒	☐	For Bayesian analysis, information on the choice of priors and Markov chain Monte Carlo settings
☒	☐	For hierarchical and complex designs, identification of the appropriate level for tests and full reporting of outcomes
☐	☒	Estimates of effect sizes (e.g. Cohen's *d*, Pearson's *r*), indicating how they were calculated

Our web collection on statistics for biologists contains articles on many of the points above.

Software and code

Policy information about availability of computer code

Data collection	No software was used for data collection.
Data analysis	The custom code used for processing and analyzing our sequence data is freely available from https://github.com/seanken/CompareSequence. FlowJo v10.5.3 software was used to process the FACS data. Cell Ranger v5 was used to process the data. For the mixture experiment, cellsnp-lite v1.2.0 and Vireo v0.5.5 was used to demultiplex, while DemuxEM v0.1.7 was used on the Perturb-seq data. The tool seqtk v1.0 was used for downsampling. Cutadapt v2.10 and SeqKit v0.15.0 were used for processing raw Ultima reads and single-end Illumina reads. DropletUtils v1.10.3 was used for some downsampling analysis. Biopython v1.79 and Python v3.7.7 were used for looking at FASTQ quality scores. R v4.0.3 and Seurat v4.0.0 were used for downstream analysis of the data while Azimuth v0.3.2 was used for annotation. AMI was calculated with aricode v1.0.0, DE genes between clusters with Presto v1.0.0, and DE between perturbations with Nebula v1.1.7. BEDTools version 2.26.0, SAMtools v1.8, and pysam v0.15.3 were used to access BAM files, and FeatureCount v1.6.2 to annotate reads. IGV v2.3.80 was used to view mapped reads. Nextflow was used to make a pipeline for reference extension. The djvdj was used to analyze TCR/BCR data. RcppML v0.3.7 was used to perform NMF. sklearn was used to fit an elastic net model. ClusterProfiler v3.18.1 and fgsea package v1.16.0 were used for enrichment analysis. For visualization we used ggplot2 v3.3.3, cowplot v1.1.1, ComplexHeatmap package v2.6.2, and the NMF v0.30.1 package.

For manuscripts utilizing custom algorithms or software that are central to the research but not yet described in published literature, software must be made available to editors and reviewers. We strongly encourage code deposition in a community repository (e.g. GitHub). See the Nature Portfolio guidelines for submitting code & software for further information.

Data

Policy information about availability of data

All manuscripts must include a data availability statement. This statement should provide the following information, where applicable:

- Accession codes, unique identifiers, or web links for publicly available datasets
- A description of any restrictions on data availability
- For clinical datasets or third party data, please ensure that the statement adheres to our policy

RNA-seq data generated in this project are available from the Gene Expression Omnibus with accession number GSE197452 and the Single Cell Portal with accession numbers SCP1759.
For the reference human genome, we used: https://cf.10xgenomics.com/supp/cell-exp/refdata-cellranger-GRCh38-1.2.0.tar.gz.
For Azimuth, we used the built in PBMC reference: https://azimuth.hubmapconsortium.org/references/#Human%20-%20PBMC.

Field-specific reporting

Please select the one below that is the best fit for your research. If you are not sure, read the appropriate sections before making your selection.

☒ Life sciences ☐ Behavioural & social sciences ☐ Ecological, evolutionary & environmental sciences

For a reference copy of the document with all sections, see nature.com/documents/nr-reporting-summary-flat.pdf

Life sciences study design

All studies must disclose on these points even when the disclosure is negative.

Sample size	No sample size calculations were performed. The number of cells per experiment was chosen to be in line with the number of cells in standard practice for 10x scRNA-seq experiments. In particular, we considered our previous work such as Ding et al. Nat Biotechnol 38, 737-746 (2020).
Data exclusions	We excluded cells filtered out by the Cell Ranger pipeline, a pre-established exclusion criteria built into the software.<br>For downstream analysis we also filtered out doublets identified by nuclei hashing or vireo (detail in Online Methods). These exclusions are relatively standard practice in the field and were applied in the course of data processing. It is not possible to pre-determine exactly which data will need to be removed without an initial analysis.
Replication	We used different sample types and methods to verify that Ultima and Illumina gave similar results by numerous metrics. Generally, our results were comparable among these experiments. Specific experiments are not replicates and further details are provided in Online Methods.
Randomization	This was not relevant because we did not do anything like select research participants or assign samples to one particular treatment. Each experiment was performed with both Illumina and Ultima sequencing. Randomization was used in sampling equal numbers of reads, UMIs, or cells as described in the Online Methods.
Blinding	This is not relevant because there is no way to blind for sequencing approach either during the experiment or analysis.

Reporting for specific materials, systems and methods

We require information from authors about some types of materials, experimental systems and methods used in many studies. Here, indicate whether each material, system or method listed is relevant to your study. If you are not sure if a list item applies to your research, read the appropriate section before selecting a response.

Materials & experimental systems

n/a	Involved in the study	
☐	☒	Antibodies
☐	☒	Eukaryotic cell lines
☒	☐	Palaeontology and archaeology
☒	☐	Animals and other organisms
☒	☐	Human research participants
☒	☐	Clinical data
☒	☐	Dual use research of concern

Methods

n/a	Involved in the study	
☒	☐	ChIP-seq
☐	☒	Flow cytometry
☒	☐	MRI-based neuroimaging

Antibodies

Antibodies used	We stained cells with CITE-seq and hashing antibodies as previously described 22 (table S4) along with a fluorescent HLA antibody (BioLegend 311415).
Validation	Hashing antibodies were used in reference 22. The HLA antibody validation is shown in vendor website. https://www.biolegend.com/en-us/search-results/alexa-fluor-488-anti-human-hla-a-b-c-antibody-2899.

Eukaryotic cell lines

Policy information about cell lines

Cell line source(s)	A375 cells (ATCC CRL-1619)
Authentication	Vendor website indicates STR authentication (https://www.atcc.org/products/crl-1619#detailed-product-information). No additional authentication was done.
Mycoplasma contamination	A375 cells used in this study tested negative for mycoplasma.
Commonly misidentified lines (See ICLAC register)	Not used.

Flow Cytometry

Plots

Confirm that:

- ☐ The axis labels state the marker and fluorochrome used (e.g. CD4-FITC).
- ☐ The axis scales are clearly visible. Include numbers along axes only for bottom left plot of group (a 'group' is an analysis of identical markers).
- ☐ All plots are contour plots with outliers or pseudocolor plots.
- ☐ A numerical value for number of cells or percentage (with statistics) is provided.

Methodology

Sample preparation	Cells were harvested, blocked in 2% FBS in PBS and stained with an anti-HLA-A,B,C FITC-conjugated antibody prior to flow cytometry.
Instrument	Sony MA900
Software	Flow cytometry data was analyzed using FlowJo v10.5.3 software.
Cell population abundance	The sorted cell population was the lowest 5% of cells based on HLA-FITC antibody binding signal.
Gating strategy	Live cells were sorted based on FSC and SSC. From this live population, mKate2+ cells were selected. Lastly, the lowest 5% of FITC+ cells was selected based on signal from an HLA-FITC antibody.

- ☐ Tick this box to confirm that a figure exemplifying the gating strategy is provided in the Supplementary Information.
